# Peripheral Developing Odontoma or Peripheral Ameloblastic Fibroodontoma: A Rare Challenging Case

**DOI:** 10.1155/2016/9379017

**Published:** 2016-02-15

**Authors:** Saede Atarbashi Moghadam, Sepideh Mokhtari

**Affiliations:** Department of Oral and Maxillofacial Pathology, Dental School of Shahid Beheshti University of Medical Sciences, Velenjak Street, Tehran 1985717443, Iran

## Abstract

Peripheral odontogenic lesions are considered to be rare within the classification of odontogenic tumors. They share the same microscopic characteristics of their central counterparts. Here, we report an ulcerated mass of the maxillary gingiva that on histopathological examination was diagnosed as peripheral developing odontoma or peripheral ameloblastic fibroodontoma. The diagnosis of this tumor is challenging and may lead to unnecessary treatment.

## 1. Case Report

**Figure 1:**
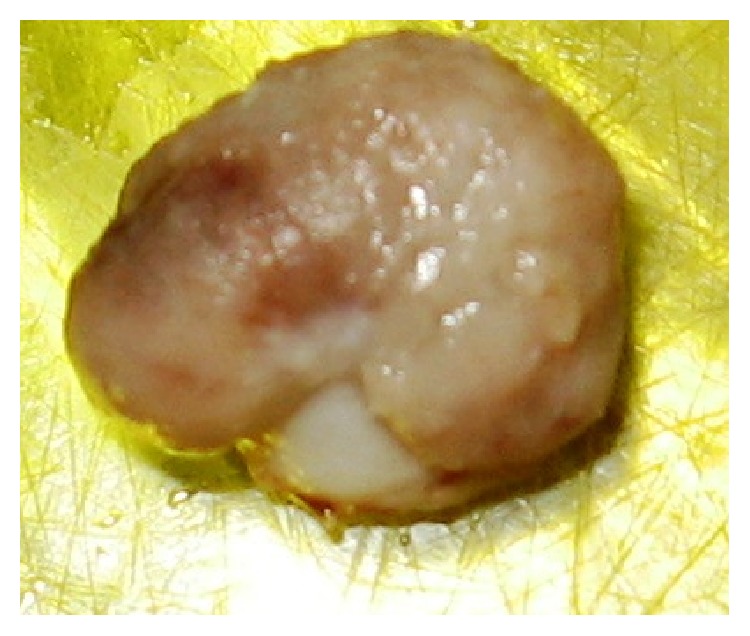
Gross of the specimen shows a nodular mass covered by ulcerated mucosa.

**Figure 2:**
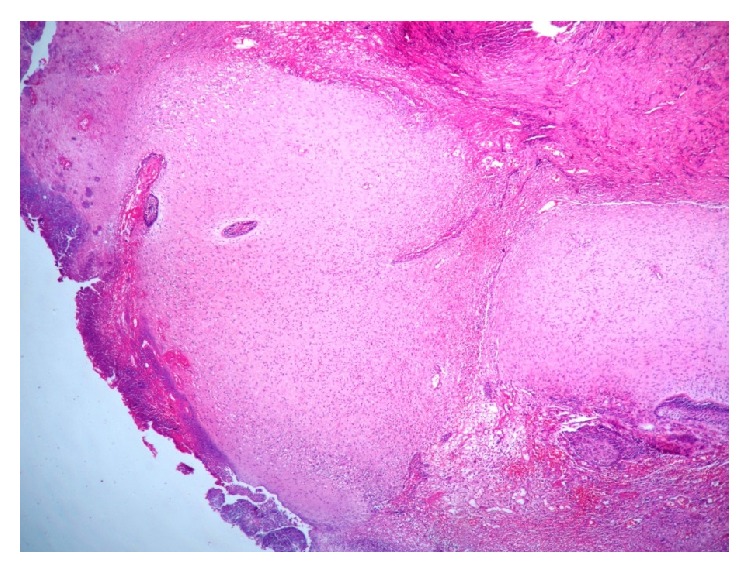
Dental papilla-like connective tissue covered by fibrinopurulent membrane with bacterial colonization (H&E, ×100).

**Figure 3:**
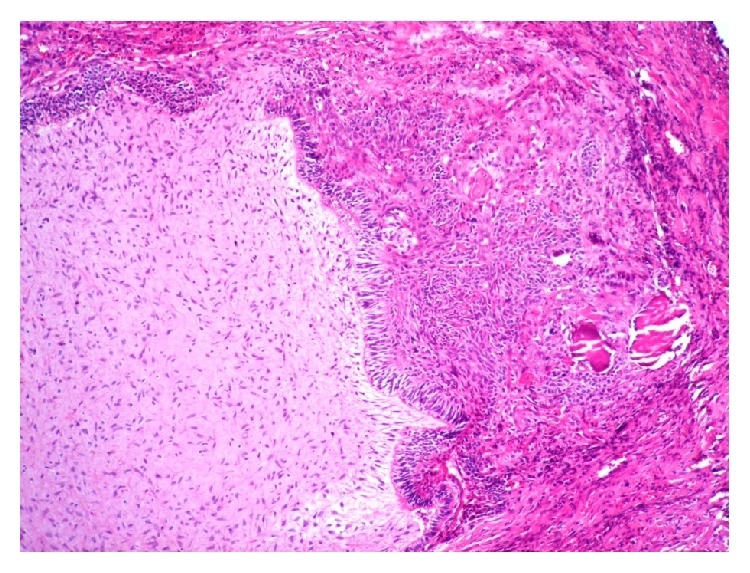
Odontogenic epithelium separated from dental papilla-like tissue with tall columnar cells (H&E, ×400). Odontogenic epithelium is also intermixed with ghost cells and calcification.

An 8-year-old healthy girl was referred to the pathology department for examination of an isolated and ulcerated soft tissue mass of the palatal gingiva in the region of right canine and deciduous first molar, measuring 0.8 × 0.8 cm with unknown duration. There was no history of trauma to the area. The periapical radiograph did not show any intrabony lesion. The mass was excised under local anesthesia with clinical diagnosis of reactive soft tissue lesion most probably pyogenic granuloma (PG) or peripheral ossifying fibroma (POF). The cut surface of the lesion was creamy solid (Figure 1). On histopathologic examination the oral epithelium was totally replaced by fibrinopurulent membrane with bacterial colonization. The underlying connective tissue demonstrated dental papilla-like structure and irregularly proliferating dental epithelium composed of stellate reticulum-like and tall columnar ameloblast-like cells. A small accumulation of ghost cells and calcification were also seen. The mineralized material was a basophilic deposition without any specific structure and more similar to foci of immature enamel or dentin. Scattered odontogenic islands resembling the islands of ameloblastic fibroma were seen in dental papilla area. This morphology was irregular and not that of an immature tooth germ (Figures 2 and 3). The lesion was diagnosed as peripheral developing complex odontoma or peripheral ameloblastic fibroodontoma. Unfortunately, no clinical image was recorded by the surgeon since the lesion was clinically diagnosed as a peripheral reactive lesion. The patient's postoperative course was normal, and there was no evidence of recurrence with 1-year follow-up period.

## 2. Discussion

Odontogenic neoplasms are categorized into peripheral and central. The relative frequency of peripheral odontogenic tumors is rare and there is no valid information of their frequency in the literature [1]. Saghravanian et al. [2] reported that 4.3% of odontogenic tumors were peripheral. In several large series, peripheral odontogenic fibroma was the most common peripheral odontogenic tumor followed by peripheral ameloblastoma [1–4]. There are some discrepancies in incidence, sex, age, and location of these tumors. This could be attributed to the differences in the sample size and the scarcity of these lesions [4]. Peripheral odontogenic tumors may be misdiagnosed clinically as common reactive soft tissue lesions in the oral cavity like pyogenic granuloma, irritation fibroma, peripheral giant cell granuloma, and peripheral ossifying fibroma [2]. Therefore, it is essential for oral professionals to be familiar with their clinical and microscopic features to prevent misdiagnosis especially with truly neoplastic odontogenic lesions.

Odontoma and ameloblastic fibroodontoma belong to mixed odontogenic tumors group and are composed of odontogenic epithelium and ectomesenchyme [5]. Some authors regard complex odontoma and ameloblastic fibroodontoma as hamartoma rather than true neoplasm. Moreover, they consider ameloblastic fibroodontoma to be a stage that goes before the complex odontoma [6]. This concept about a peripheral developing odontoma or peripheral ameloblastic fibroodontoma inserts diagnostic information about this entity to prevent extensive surgery.

The etiology of odontogenic tumors is indefinite and their source is the remnants of dental lamina that reside in the gingiva (rests of Serres) and develop lingually to those of the primary teeth [5]. Therefore, many reports mention that peripheral odontoma occurs in palatally or lingually position [7–9]. As in this case, maxillary gingiva is involved more than mandibular gingiva [7–9]. Peripheral developing odontoma has been found entirely in children and congenital cases have also been reported [8]. Ide et al. [10] found that the mean age of patients with peripheral developing odontoma was 6.6 years which is 10 years younger than the mean age of patients with central odontoma. This lesion is reported in both boys and girls and does not show sex predilection [9].

The clinical presentation of this case was similar to PG and POF because of an ulcerated surface and the gingival involvement. In the case described by Kintarak et al. [7], focal fibrous hyperplasia and POF were described as the first differential diagnosis.

The presence of dental tissue outside the alveolar process may be associated with ectopic neural crest cells that are still able to differentiate into tooth germ [8]. It is difficult to distinguish ameloblastic fibroodontoma from a developing complex odontoma. Some investigators believe the two tumors to be part of a range in children [5]. Thus, the first step is a nonneoplastic type of ameloblastic fibroma (consisting of odontogenic epithelium and odontogenic ectomesenchyme) in which dentin and then enamel are formed. At the end stage, it forms a complex odontoma almost entirely consisting of dental hard tissues [6]. Therefore, different morphological features that are found in the cases of peripheral odontoma depend on the developmental stage of tooth germ [8]. In the present case, hard tissue formation was minimal and the specimen was easily cut for microscopic preparation.

The treatment choice is conservative surgical excision [5]. Recurrence of this lesion has not been documented [7]. Peripheral odontoma has a limited growth potential. However, if this lesion is not surgically excised in the early developmental phase, it may erupt into the oral cavity. The eruptive mechanism of this lesion remains uncertain and it appears to be different from tooth eruption because of the lack of periodontal attachment in odontoma [11].

## 3. Conclusion

Peripheral odontoma and peripheral ameloblastic fibroodontoma are exceedingly rare benign odontogenic lesions that are treated by conservative excision. The proper diagnosis of these lesions is necessary to avoid confusion with true neoplasms especially odontogenic tumors and prevent extensive surgery. Therefore, it is essential for oral pathologists to be familiar with clinical and microscopic characteristics of peripheral odontoma.
